# Behavioural evidence for segments as subordinate units in Chinese spoken word production: The form-preparation paradigm revisited

**DOI:** 10.1371/journal.pone.0225718

**Published:** 2019-11-27

**Authors:** Jie Wang, Andus Wing-Kuen Wong, Yiu-Kei Tsang, Suiping Wang, Hsuan-Chih Chen

**Affiliations:** 1 Department of Psychology, The Education University of Hong Kong, Hong Kong S.A.R., China; 2 Nam Shan Psychology Laboratory, Department of Social and Behavioural Sciences, City University of Hong Kong, Hong Kong S.A.R., China; 3 Department of Education Studies, Hong Kong Baptist University, Hong Kong S.A.R., China; 4 School of Psychology, South China Normal University, Guangzhou, China; 5 Department of Psychology, The Chinese University of Hong Kong, Hong Kong S.A.R., China; Universita degli Studi di Milano-Bicocca, ITALY

## Abstract

It is widely acknowledged that phonemic segments are primary phonological units, processed serially, in spoken word production of Germanic languages. However, evidence for a behavioural effect of single-segment overlap on Chinese spoken word production is lacking. The current study adopted the form-preparation paradigm to investigate the effects of segment predictability and segment repetition separately, which were mixed in previous studies. Native Mandarin Chinese speakers named pictures in the following conditions: predictable, unpredictable, and no segment repetition. Different positions in words (i.e., the onset and the rhyme) were examined at the same time. Results revealed a facilitation effect of onset predictability masked by an inhibition tendency of onset repetition, indicating Chinese speakers’ ability to prepare the predictable onset. In contrast, rhyme predictability showed a non-significant effect. This pattern of results did not change no matter whether the conditions of unpredictable onset repetition and unpredictable rhyme repetition were mixed in the same context (Experiment 1) or extracted from different blocked contexts (Experiment 2). The finding provides essential support to the claim that phonemic segments are functionally engaged in Chinese spoken word production, and thus adds original evidence to the universal aspect of spoken word production.

## Introduction

Language production plays an essential role in human daily life. Revealing the related mechanisms will advance our understanding of human cognition. To produce a word in speaking, its phonological word form must be constructed at the planning stage [1, 2]. This process is called word-form encoding. In recent decades, an increasing number of studies have been conducted to investigate the word-form encoding process in language production. Spoken words consist of syllables (e.g., /ba/, /ma/), while syllables can be further divided into phonemic segments (e.g., /b/, /a/, /m/). What roles these different phonological units play in the planning stage remains one of the fundamental issues in language production.

The form-preparation paradigm [3, 4] has been widely used to address the above-mentioned issue. This paradigm requires participants to produce spoken words in a homogeneous context where all the target words share a phonological component (e.g., the same onset consonant, *hut*, *heks*, *hiel*—“hut”, “witch”, “heel”) and a heterogeneous context with unrelated target words. If the participants’ naming latencies are shorter in the homogeneous context than in the heterogeneous context, researchers believe that the shared phonological components can be prepared in advance by the participants and thus consist of selectable planning units [5]. An onset-preparation effect has been found in multiple alphabetic languages (Dutch: [4]; English: [6, 7]; French: [8]; see [9, 10] for other types of onset effects in alphabetic languages). However, previous form-preparation studies in Chinese and Japanese failed to show a similar onset effect, but instead found robust effects of sharing atonal syllables in Chinese [11, 12] and facilitation effects of sharing moras in Japanese [13].

O’Seaghdha et al. [7] proposed a proximate units principle to account for the discrepant results in the mentioned studies conducted in different languages. The “proximate units” refer to the phonological units that speakers can directly select below the word level, and these units are assumed to be language-specific, such as segments in English, atonal syllables in Chinese, and moras in Japanese. Note that it does not mean that subordinate phonological units are not engaged in spoken word production at all. For example, sub-syllabic effects (i.e., body or rhyme effects) have been found in Chinese with different paradigms of word production (form-preparation paradigm: [12]; picture-word interference paradigm: [14–16]; masked priming paradigm: [17]). Further evidence suggests that syllabic encoding precedes sub-syllabic encoding in Chinese spoken word production [18–22].

Moreover, although manipulation on more than one segment has been shown sufficient to generate behavioural effects on Chinese spoken word production [12, 14–17], no behavioural difference has been observed under manipulation on single segment in Chinese (see an exceptional onset effect in [23], where stimuli were presented in Pinyin syllables). So far the Chinese onset effect has only been demonstrated with electrophysiological measures, which presumably are more sensitive than behavioral ones [24, 25]. How to further investigate the segmental encoding process in Chinese has become a critical issue in exploring the universal and/or language-specific aspects of spoken word production. In other words, even if phonemic segments are universal phonological units, it remains to be determined whether or not they are processed in the same manner at the proximate and the subordinate levels.

Meyer [4] found in Dutch that the form-preparation effect occurred when the onset was shared but not when the rhyme was shared, suggesting that segments as proximate units are processed serially from the beginning of the word to its end. In contrast, little evidence is available to show how single segments could be processed when they are subordinate units, due to the difficulty in finding a behavioural effect of subordinate units. Fortunately, a study of Breining, Nozari, and Rapp [26] provided us inspirations. They designed another type of homogeneous context by distributing the segmental overlap across different positions in words (e.g., *cat*, *mat*, *cot*, *cap*, *map*, *mop*). After the predictability of word-initial segments was removed, mere segment repetition inhibited (not facilitated) English-speaking participants’ naming responses. It seems that the observed facilitation effect in previous form-preparation studies is a net effect of two opposing forces: a facilitation effect of segment predictability and an inhibition effect of segment repetition. The facilitation effect can be observed because it is stronger than the inhibition effect. Do these two opposite effects still exist when segments are subordinate units?

Assuming that the facilitation effect of segment predictability and the inhibition effect of segment repetition also exist in Chinese spoken word production, we may infer that the null onset effect is a result of the facilitation effect (of onset predictability) cancelling out the inhibition effect (of onset repetition). It is reasonable to expect a weaker facilitation effect of onset predictability when segments are subordinate units than when they are proximate units, and this could be the reason why the facilitation effect fails to outweigh the inhibition effect. Alternatively, these two opposite effects may not exist when segments are subordinate units. Hence, the current study aimed to investigate whether onset predictability can facilitate Chinese speakers’ naming responses in the form-preparation paradigm. As a comparison, we also examined the effect of rhyme predictability.

To examine the effect of segment predictability, we first replicated the design of Breining, et al. [26] in Experiment 1. Native speakers of Mandarin Chinese were asked to name individually presented pictures with pre-designated names in different contexts. We had typical homogeneous contexts in which the same onset or the same rhyme was repeated predictably, an overlap-distributed context in which onset repetition or rhyme repetition occurred in some trials unpredictably (e.g., *巢* /chao2/, *锤* /chui2/, *柜* /gui4/, *糕* /gao1/), as well as a heterogeneous context without segment repetition. Traditional form-preparation effects are calculated by subtracting picture naming latencies (i.e., RTs) in the heterogeneous context from those in the homogeneous context (RT_Predictable segment repetition_—RT_No segment repetition_), which are actually net effects of two factors—segment predictability (RT_Predictable segment repetition_—RT_Unpredictable segment repetition_) and segment repetition (RT_Unpredictable segment repetition_—RT_No segment repetition_). In the current study, we aimed to directly investigate the effect of segment predictability through RT comparisons between the conditions of predictable and unpredictable segment repetition. Importantly, we extracted trials with immediate onset repetition or rhyme repetition after the preceding trials from the overlap-distributed context, so as to investigate the effects of onset predictability (RT_Predictable onset repetition_—RT_Unpredictable onset repetition_) and rhyme predictability (RT_Predictable rhyme repetition_ vs. RT_Unpredictable rhyme repetition_) respectively.

One possible limitation of using the overlap-distributed context in Experiment 1 is that the conditions of unpredictable onset repetition and unpredictable rhyme repetition were mixed together in the same context. The effects of onset repetition and rhyme repetition might contaminate each other if segmental overlap between two picture names with the presence of intervening items also inhibits naming of the second picture (see examples of semantic interference with intervening items in [27, 28]). Specifically, the naming response to Picture n could be influenced by not only the segmental overlap between Pictures n and n-1, but also the segmental overlap between Pictures n and n-2, Pictures n and n-3, and so forth. In the overlap-distributed context, Picture n might share the same onset with Picture n-1, but the same rhyme with Picture n-2. Thus, the effect of a certain segmental overlap between consecutive picture names (i.e., immediate segment repetition) could be confounded by the other type of segmental overlap in the sequence of naming. Hence, we designed two separate contexts for unpredictable onset repetition and unpredictable rhyme repetition in Experiment 2. For example, in the picture set of *车* /che1/, *愁* /chou2/, *水* /shui3/, *勺* /shao2/, two different onset segments /ch/ and /sh/ were shared among the picture names but no rhyme was shared. In this way, onset repetition between consecutive picture names was still unpredictable and at the same time isolated from any confounding effect of rhyme repetition. The context of unpredictable rhyme repetition was designed in a similar manner.

## Experiment 1

In this experiment, we adopted the overlap-distributed context of Breining, et al. [26] where onset repetition and rhyme repetition were mixed and occurred unpredictably. Trials with immediate onset repetition or rhyme repetition after the preceding trials were extracted separately to constitute the conditions of unpredictable onset repetition and unpredictable rhyme repetition.

### Method

#### Participants

The current study was approved by Survey and Behavioural Research Ethics Committee, The Chinese University of Hong Kong. Thirty-two students (5 males; age = 22.0 ± 2.9 years) from The Chinese University of Hong Kong participated in Experiment 1 with monetary rewards (30 HKD per participant). They were native Mandarin speakers from Mainland China, and had stayed in Hong Kong for 0.5 year on average (SD = 0.9 year). All participants had learned English as their second language, and some of them also speak other Chinese dialects (e.g., Cantonese). They all had normal or corrected-to-normal vision, and were neurologically healthy. Written consent was obtained from each participant before the experiment.

#### Stimuli and apparatus

Sixteen white-on-black line drawings were selected as stimuli. Their names were monosyllabic Mandarin Chinese words consisting of different combinations of four onsets /g/, /h/, /sh/, /ch/ and four rhymes /e/, /ui/, /ao/, /ou/ (e.g., *车* /che1/, meaning “*car”*). Table 1 shows examples of how these pictures were grouped to generate four different types of contexts: 1) predictable onset repetition (PO): all the four picture names within a set shared the same onset; 2) predictable rhyme repetition (PR): all the four picture names within a set shared the same rhyme; 3) distributed segmental overlap (O: unpredictable onset repetition; R: unpredictable rhyme repetition): both onset overlap and rhyme overlap existed in different pairs of picture names within a set; and 4) unrelated control (U): no onset segment or rhyme was shared among the four picture names. There were four picture sets for each type of contexts (see S1 Text). Note that in the context of distributed segmental overlap, a picture (e.g., *锤* /chui2/ “*hammer*”) could be preceded by another picture with onset overlap (e.g., *巢* /chao2/ “*nest*”) or rhyme overlap (e.g., *柜* /gui4/ “*cabinet*”) in their names. Trials with immediate onset repetition or rhyme repetition after the preceding trials were extracted separately from the overlap-distributed context, constituting the O and the R conditions respectively. For example, a trial with a picture named *锤* /chui2/ belonged to the O condition if preceded by a trial with *巢* /chao2/, or the R condition if preceded by a trial with *柜* /gui4/. Therefore, the four types of contexts generated five different conditions, called priming conditions in the following text.

**Table 1:** Contexts and priming conditions in Experiments 1 and 2. In the context of distributed segmental overlap in Experiment 1, a picture (e.g., *锤* /chui2/ “*hammer*”) could be preceded by another picture with onset overlap (e.g., *巢* /chao2/ “*nest*”) or rhyme overlap (e.g., *柜* /gui4/ “*cabinet*”) in their names, so that onset repetition and rhyme repetition were mixed and unpredictable. Trials with immediate onset repetition or rhyme repetition after the preceding trials constituted the priming condition of unpredictable onset repetition (O) or unpredictable rhyme repetition (R).

	Context	Picture set example	Segment repetition	Segment predictability	Overlap position	Overlap distribution	Priming condition
Exp. 1	Predictable onset repetition	*车* /che1/, *巢* /chao2/, *锤* /chui2/, *愁* /chou2/	+	+	Onset	Blocked	PO
	Predictable rhyme repetition	*车* /che1/, *蛇* /she2/, *盒* /he2/, *鸽* /ge1/	+	+	Rhyme	Blocked	PR
	Distributed segmental overlap	*巢* /chao2/, *锤* /chui2/, *柜* /gui4/, *糕* /gao1/	+	-	Onset/Rhyme	Distributed	O/R
	Unrelated control	*车* /che1/, *猴* /hou2/, *柜* /gui4/, *勺* /shao2/	-	-	---	---	U
Exp. 2	Predictable onset repetition	*车* /che1/, *巢* /chao2/, *锤* /chui2/, *愁* /chou2/	+	+	Onset	Blocked	PO
	Predictable rhyme repetition	*车* /che1/, *蛇* /she2/, *盒* /he2/, *鸽* /ge1/	+	+	Rhyme	Blocked	PR
	Unpredictable onset repetition	*车* /che1/, *愁* /chou2/, *水* /shui3/, *勺* /shao2/	+	-	Onset	Blocked	O
	Unpredictable rhyme repetition	*蛇* /she2/, *盒* /he2/, *锤* /chui2/, *柜* /gui4/	+	-	Rhyme	Blocked	R
	Unrelated control	*车* /che1/, *猴* /hou2/, *柜* /gui4/, *勺* /shao2/	-	-	---	---	U

Eighteen native Mandarin speakers who did not participate in the main experiments were asked to rate both the semantic relatedness and the visual similarity of all possible picture pairs on a 5-point scale (1 denoting *not at all related/similar*, 5 denoting *highly related/similar*). Pairs of filler items with high semantic relatedness or visual similarity were added into the rating list and mixed with target pairs. For example, *花* “*flower*” and *草* “*grass*” were one pair of filler items with high semantic relatedness. The filler items scored 4.3 ± 0.3 on semantic relatedness and 3.7 ± 0.5 on visual similarity. The average scores of semantic relatedness were 1.5 ± 0.5, 1.5 ± 0.5, 1.5 ± 0.5, and 1.5 ± 0.4, for picture pairs in the predictable-onset, predictable-rhyme, overlap-distributed, and unrelated contexts; and the average scores of visual similarity were 1.3 ± 0.3, 1.3 ± 0.3, 1.3 ± 0.3, and 1.3 ± 0.3. Besides the relatively low semantic relatedness and visual similarity, the picture names within a set were unrelated in orthographic forms.

The pictures were presented with E-Prime 2.0 software, expanding 6 cm × 6 cm (approximately 5° × 5° in visual angle) on the computer screen, whose refresh rate was 60 Hz. The onset of participants’ naming response was detected by a serial response-box with a microphone.

#### Design and procedure

There were 16 picture sets (4 contexts × 4) in total. Each set of pictures were named cyclically (5 cycles) in a separate block. The order of the pictures were pseudorandomized so that two consecutive pictures were always different. The four blocks of each context were presented consecutively, forming a “superblock” [6]. The order of the blocks within a superblock as well as the order of superblocks were counterbalanced across participants. Among the thirty-two participants, each type of superblocks appeared eight times in each ordinal position, and the four blocks within a superblock were reverse counterbalanced.

In the beginning of each experiment, participants were asked to learn the names of the 16 pictures in 16 practice trials (i.e., one practice trial for each picture), during which naming errors were corrected. After that, they performed picture naming in the experimental blocks. In each block, participants were first shown the whole set of four pictures to appear in this block and then pressed a key to start. In each experimental trial, a 500-ms white fixation at the center of the black screen and a 500-ms blank were first presented. The picture was then presented at the center for 2000 ms or until a naming response was detected by the voicekey. Participants were required to name the picture aloud as accurately and quickly as possible. The inter-trial interval varied randomly from 1500 to 2500 ms. Short breaks were allowed between blocks. The whole procedure lasted for approximately 30 minutes.

#### Data analyses

Naming latencies were inverse transformed (-1000/RT) to approximate the normal distribution and submitted to linear mixed-effect modeling (LMEM; [29, 30]) in R Version 3.4.3 [31]. *p* values were calculated with Satterthwaite approximation via *lmerTest* package [32]. Priming condition (cond), cycle (cyc), and their interaction were fixed effects, while participants and items were random effects. The random effects structure included by-participant and by-item random intercepts, as well as by-participant and by-item random slopes for priming condition (random slopes for other fixed effects were not included, since models with these parameters failed to converge). In addition, superblock order (ord) was also entered as a fixed effect to control for possible confounding. The formula for this model was [invRT ~ cond*cyc + ord + (1 + cond | participant) + (1 + cond | picture)]. More details of the analyses can be found in S2 Text. The error rates were not analyzed in detail, because errors were very rare (below 1%).

### Results

**Fig 1:**
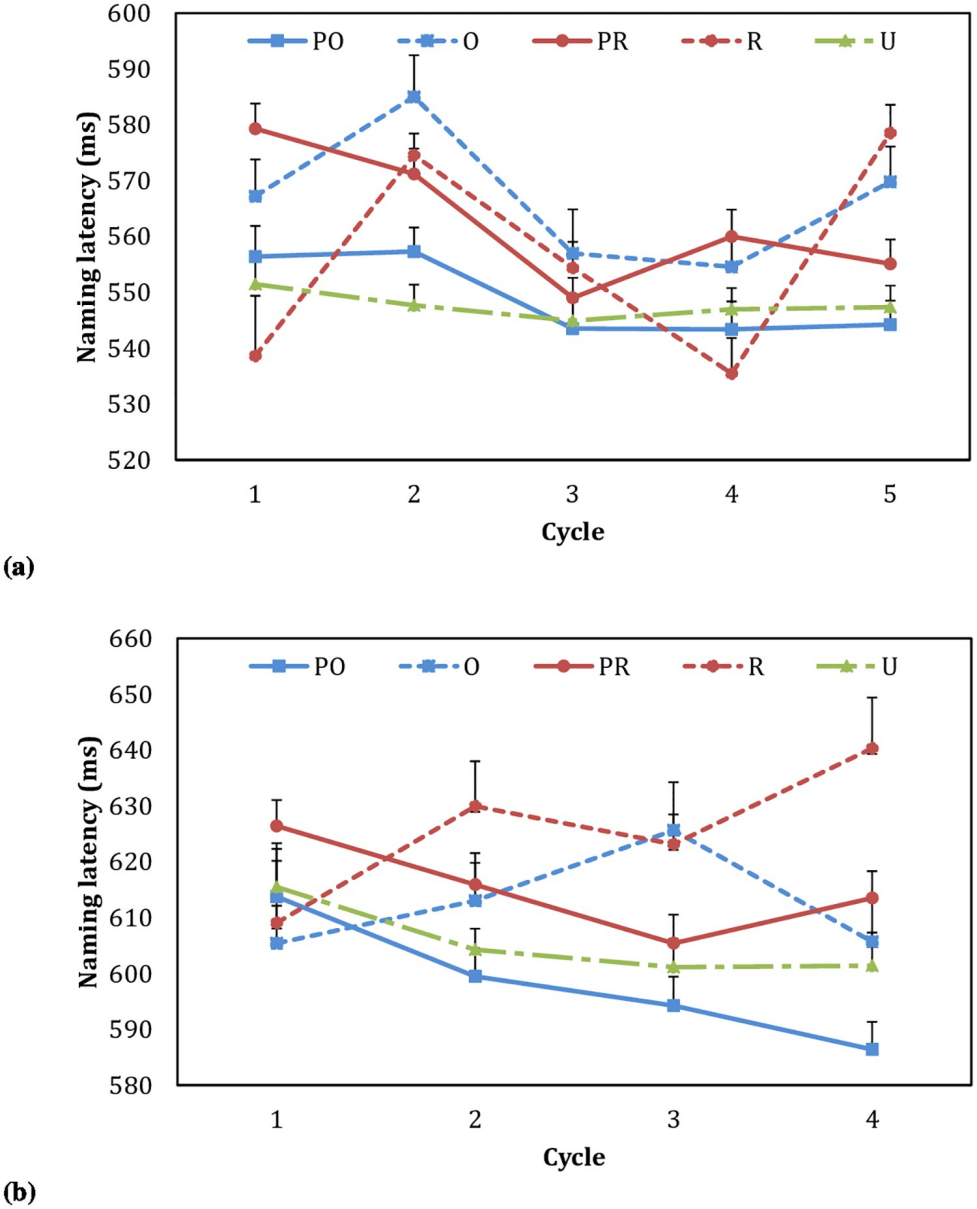
Mean naming latencies at each cycle in Experiments 1 (a) and 2 (b). Standard errors of the means shown as the error bars are corrected for repeated measures [34]. PO, predictable onset repetition; PR, predictable rhyme repetition; O, unpredictable onset repetition; R, unpredictable rhyme repetition; U, unrelated control. In Experiment 1, the O and the R conditions were mixed in the context of distributed segmental overlap, where a picture (e.g., *锤* /chui2/ “*hammer*”) could be preceded by another picture with onset overlap (e.g., *巢* /chao2/ “*nest*”) or rhyme overlap (e.g., *柜* /gui4/ “*cabinet*”) in their names.

Trials with incorrect or no naming responses (0.5%), voicekey mistriggers or technical errors (3.0%), or extreme latencies (exceeding 2.5 SD of individual mean or item mean, 2.9%) were excluded from naming latency analyses. Fig 1a displays the mean naming latencies at each cycle. Data from Cycle 1 were excluded from LMEM analyses, because context effects are typically found after the first cycle (e.g., [26, 33]). In the LMEM analyses (see S2 Text for details), likelihood ratio tests demonstrated that adding the interaction of priming condition and cycle to the model did not improve model fit significantly (*χ*^2^_(12)_ = 20.35, *p* = 0.061) but adding the factor priming condition did (*χ*^2^_(4)_ = 11.27, *p* = 0.024). Thus, the following model was adopted: [invRT ~ cond + cyc + ord + (1 + cond | participant) + (1 + cond | picture)]. Below we report regression coefficients (*b*), standard errors (*SE*), and *p* values for the factor priming condition in this model. First, relative to the U condition, a null effect was found for the PO condition (*b* = 0.005, *SE* = 0.021, *p* = 0.825), while a significant inhibition effect was found for the PR condition (*b* = 0.046, *SE* = 0.018, *p* = 0.014). Second, onset repetition generated a significant inhibition effect (O vs. U: *b* = 0.058, *SE* = 0.022, *p* = 0.022), while rhyme repetition generated a marginally significant inhibition effect (R vs. U: *b* = 0.044, *SE* = 0.024, *p* = 0.072). Third, onset predictability generated a significant facilitation effect (PO vs. O: *b* = -0.053, *SE* = 0.024, *p* = 0.039), while rhyme predictability generated a null effect (PR vs. R: *b* = 0.002, *SE* = 0.019, *p* = 0.915).

### Discussion

The comparisons between the traditional homogeneous blocks versus heterogeneous blocks in the current experiment demonstrated a null effect of predictable onset repetition (PO vs. U) and an inhibition effect of predictable rhyme repetition (PR vs. U), consistent with previous findings [7, 11, 12, 35, 36]. Both onset repetition and rhyme repetition showed a trend of inhibiting the naming responses. When we further compared the conditions of predictable segment repetition and unpredictable segment repetition, a facilitation effect of onset predictability (PO vs. O) was uncovered in contrast to a null effect of rhyme predictability (PR vs. R).

However, the results of Experiment 1 should be interpreted with caution. As mentioned in the Introduction section, the two conditions of unpredictable onset repetition and unpredictable rhyme repetition were extracted from the same context in Experiment 1 and thus could confound each other. Hence, an improved design was adopted in Experiment 2, where these two conditions were blocked to prevent the potential confounding.

## Experiment 2

In this experiment, we replaced the overlap-distributed context with two different contexts of unpredictable onset repetition and unpredictable rhyme repetition. All the other contexts were the same as in Experiment 1.

### Method

#### Participants

The current study was approved by Survey and Behavioural Research Ethics Committee, The Chinese University of Hong Kong. Thirty native Mandarin speakers (4 males; age = 20.2 ± 2.7 years) from South China Normal University participated in Experiment 2 with monetary rewards (20 RMB per participant). All participants had learned English as their second language, and some of them also speak other Chinese dialects (e.g., Cantonese). They all had normal or corrected-to-normal vision, and were neurologically healthy. Written consent was obtained from each participant before the experiment.

#### Stimuli and apparatus

The same stimuli and apparatus were used as in Experiment 1. As shown in Table 1, besides the contexts of PO, PR, and U, two new contexts were included: 1) unpredictable onset repetition (O): two different onsets were shared among the four picture names and no rhyme was shared; 2) unpredictable rhyme repetition (R): two different rhymes were shared among the four picture names and no onset was shared. The major difference from Experiment 1 was that the O and the R conditions were extracted from the two new contexts respectively (they were extracted from the same overlap-distributed context in Experiment 1). Consequently, the same priming conditions were generated in Experiment 2—PO, PR, O, R and U.

Based on the rating results of the eighteen native Mandarin speakers (the same group of people as in Experiment 1), the average scores of semantic relatedness were 1.4 ± 0.4 and 1.5 ± 0.5 for picture pairs in the unpredictable-onset and unpredictable-rhyme contexts, and the average scores of visual similarity were 1.2 ± 0.3 and 1.3 ± 0.3 respectively, comparable to those of the other contexts. Besides, the picture names within a set were unrelated in orthographic forms.

#### Design and procedure

There were 20 picture sets (5 contexts × 4) in Experiment 2. Each set of pictures were named cyclically (4 cycles) in a separate block. The order of the pictures were pseudorandomized so that two consecutive pictures were always different. The same type of blocks were presented consecutively in a superblock as in Experiment 1. The order of the blocks within a superblock as well as the order of superblocks were counterbalanced across participants. Among the thirty participants, each type of superblocks appeared six times in each ordinal position, and the four blocks within a superblock were reverse counterbalanced. The procedure of the experimental trials and the practice part were the same as in Experiment 1.

#### Data analyses

The same method of data analyses was applied as in Experiment 1.

### Results

Trials with incorrect or no naming responses (0.8%), voicekey mistriggers or technical errors (2.8%), or extreme latencies (exceeding 2.5 SD of individual mean or item mean, 5.5%) were excluded from naming latency analyses. Fig 1b displays the mean naming latencies at each cycle. Data from Cycle 1 were excluded from further analyses. In the LMEM analyses, the same simplified model was adopted as in Experiment 1 (likelihood ratio tests: the interaction of priming condition and cycle, *χ*^2^_(8)_ = 8.75, *p* = 0.364; priming condition, *χ*^2^_(4)_ = 14.00, *p* = 0.007). Relative to the U condition, null effects were found for the PO and the PR conditions (PO: *b* = -0.030, *SE* = 0.019, *p* = 0.133; PR: *b* = 0.029, *SE* = 0.020, *p* = 0.165). Besides, onset repetition showed a non-significant trend of inhibition (O vs. U: *b* = 0.016, *SE* = 0.021, *p* = 0.464), while rhyme repetition showed a significant inhibition effect (R vs. U: *b* = 0.055, *SE* = 0.023, *p* = 0.025). In addition, the facilitation effect of onset predictability was significant (PO vs. O: *b* = -0.046, *SE* = 0.022, *p* = 0.045), but the effect of rhyme predictability was non-significant (PR vs. R: *b* = -0.026, *SE* = 0.021, *p* = 0.218).

### Discussion

Before discussing the results of the newly designed conditions, we can first look at those of the traditional homogeneous and heterogeneous conditions. While the RT difference between the PO and the U conditions remained non-significant, the inhibition effect on naming latencies in the PR condition relative to the U condition, which was significant in Experiment 1, became non-significant in Experiment 2. Still, Fig 1b shows a consistent inhibition trend of predictable rhyme repetition across cycles. The reason why its significance level fluctuated across experiments in the current study is unclear and requires further investigation. Nevertheless, the two important effects of our main interest demonstrated the same pattern across experiments, i.e., a significant onset predictability effect and a non-significant rhyme predictability effect.

The major difference in design between the two experiments is that the two conditions of unpredictable segment repetition were mixed in the same context (i.e., the overlap-distributed context) in Experiment 1 but were blocked (in the unpredictable-onset/rhyme context) in Experiment 2. In the overlap-distributed context, each picture shared the same onset with a second picture, the same rhyme with a third picture, and was unrelated to the fourth picture. In the unpredictable-onset/rhyme context, each picture only shared the same onset/rhyme with a second picture and was unrelated to the other pictures. Hence, segmental overlap occurred more frequently in the overlap-distributed context than in the unpredictable-onset/rhyme context. Recall that we mentioned in the Introduction section that the naming response to Picture n could be influenced by not only the segmental overlap between Pictures n and n-1, but also the segmental overlap between Pictures n and n-2, Pictures n and n-3, and so forth. If segmental overlap with intervening items also generates an inhibitory effect and this effect is cumulative, picture naming responses in a context with higher density of segmental overlap should be inhibited to a larger extent. Specifically, we would expect to see larger inhibition effects in the O and the R conditions in Experiment 1 than in Experiment 2. But this is not what we observed.

In Experiment 1, we found a significant RT difference between the O and the U conditions as well as a marginally significant RT difference between the R and the U conditions. In Experiment 2, the inhibition effect became non-significant in the O condition but significant in the R condition. These changes were only partly consistent with the above prediction, since the inhibition effect in the R condition was more significant in Experiment 2 than in Experiment 1. Therefore, the density of segmental overlap (i.e., the degree of phonological overlap) did not reliably moderate the inhibition effect of segment repetition. Hence, the effect of segmental overlap with intervening items was at best minimal in the current task, and the O/R conditions were similar in nature across experiments. Possible accounts for the inhibition tendency of segment repetition will be discussed in General Discussion.

The null effect of segmental overlap with intervening items also justified our comparisons between the PO/PR and the O/R conditions. Our predictability effect was obtained by subtracting the naming latency in the O/R condition from that in the PO/PR condition. Here a hidden assumption is that the inhibition effects of segment repetition were comparable between the two conditions so that we could eliminate the inhibition effect through such subtraction. Since the null effect of segmental overlap with intervening items ruled out the possibility that a context with higher density of segmental overlap generated a larger inhibition effect than a context with lower density of segmental overlap, the higher overlap density in the PO/PR condition (onset/rhyme repetition occurred in every trial) than that in the O/R condition would not undermine the above assumption. Hence, the RT difference between the PO/PR and the O/R conditions should be taken as the effect of segment predictability. Specifically, a facilitation effect of onset predictability was consistently observed in Experiments 1 and 2, while no significant effect was shown by rhyme predictability. Possible accounts for the onset predictability effect will be discussed in General Discussion.

## General discussion

In the current form-preparation study, segment repetition showed a trend of inhibiting Chinese spoken word production, while a facilitation effect was found for onset predictability. Note that the null onset effect in previous Chinese form-preparation studies [7, 11, 12, 36] was replicated in our PO and U conditions. Importantly, our results demonstrated for the first time that such a null effect might be due to the facilitation effect of onset predictability being masked by the inhibition tendency of onset repetition.

### Possible accounts for the inhibition tendency of segment repetition

Breining and colleagues [26] interpreted the inhibition effect of segmental overlap in their study as the result of incremental learning [37] during lexical-segmental mapping. According to their explanation, the links between lexical entries and their constituent segments are bidirectional. When a speaker plans to produce a word, other form-related words also get activated through the feedback from the shared segment(s). The production of the target word will strengthen the connections between the target word and its segments while weakening the connections between the form-related words and their segments (i.e. the competitive learning process), causing an inhibition effect when one of the form-related words is the next target. In the case of Chinese spoken word production, evidence suggests that syllables are directly connected to word nodes while segments are connected to syllables as subordinate phonological units [7, 18, 38]. According to the competitive learning account, bidirectional links could exist in “lexical-syllabic mapping” as well as “syllabic-segmental mapping”, and the competitive learning process might happen during “syllabic-segmental mapping”.

Other than the competitive learning account, Howard and colleagues [28] proposed that lexical entries are connected with each other through lateral inhibitory links. When a target word is activated together with its form-related competitors, it receives inhibition from the co-activated competitors. Those competitors that have been produced recently are more active and generate stronger inhibition. Therefore, producing a form-related competitor in the preceding trial generates an inhibition tendency on target word production, relative to an unrelated control.

Moreover, word production models against the assumption of interactive activation between the lexical and the segmental levels (e.g., the WEAVER++ model, [5]) may argue that the inhibition tendency of segment repetition could originate from outside the production system. For example, the WEAVER++ model could attribute the inhibition tendency to an increased workload for the self-monitoring system. Unfortunately, our current finding could not tell apart the above accounts, and further studies are needed. But this will not undermine our discussion below.

### The suspend-resume account and the attentional account for the predictability effect

The WEAVER++ model proposed a suspend-resume mechanism [5, 38, 39] to account for previous findings of the form-preparation effects. When the word-initial component is predictable in a homogeneous context, the phonological encoding process proceeds as far as possible at the preparation stage. The representation resulting from the predictable component is assumed to be buffered, and this suspended encoding process can resume after more information is available (i.e., after the stimulus onset). If the predictable component contains phonological units that are processed serially, this buffering mechanism will shorten speakers’ naming latency, well explaining the behavioural facilitation effects of predictable proximate units.

To account for the null onset effect in previous Chinese studies, the Chinese version of WEAVER++ model [38] incorporated the proximate units principle and proposed that atonal syllables as proximate units are serially encoded while segments within a syllable are subsequently specified in parallel. Even if Chinese speakers prepare the predictable onset in the buffer, it takes no less time to select other segments within the syllable, resulting in no behavioural facilitation. In the current study, however, onset predictability did facilitate Chinese speakers’ naming responses, but the effect was too weak to outweigh the inhibition tendency of onset repetition. While this onset facilitation effect agrees with the assumption of Roelofs [38] that Chinese speakers are aware of and oriented to the predictability of segments, the lack of a similar facilitation effect of rhyme predictability seems hard to reconcile with the view of parallel segmental processing.

Another account for the form-preparation effect comes from O’Seaghdha and Frazer [40]. After introducing an exception item into the homogeneous context (called “variable” homogeneous context, e.g., *beach*, *gate*, *gear*, *gown*), O’Seaghdha and Frazer found that English-speaking participants first responded more slowly to the exception item (Block 1), relative to unrelated control in the heterogeneous context, but eventually showed a trend of preparation (Block 2). At the same time, participants’ responses to the consistent items (i.e., items other than the exception one) were faster than unrelated control in both blocks. These results contradicted the prediction of the suspend-resume account that the existence of an exception item would prevent participants from storing a constant representation in the buffer and thus eliminate the facilitation effect. Hence, O’Seaghdha and Frazer proposed that form preparation should be understood as an attentional process, which may pre-activate accessible units instead of pre-entering a partial representation into the buffer of the production system. Specifically, participants first attended to the onset shared by the consistent items but not to the exception item in Block 1. After sufficient task experience (Block 2), participants were able to develop “dual attention to a majority component and an exception”.

This attentional account adds complication to the interpretation of the current onset predictability effect, which was obtained by subtracting the naming latency in the O condition from that in the PO condition. Since the attentional account allows participants to attend to more than one phonological unit, it is theoretically possible that our participants in both experiments not only prepared the single predictable onset in the PO condition but also prepared the two equiprobable onsets by dual attention in the O condition. The possible effect of this “partial onset predictability” in the O condition means that onset predictability might not facilitate the naming latencies in an all-or-none manner. In other words, the degree of phonological overlap (sharing one same onset, sharing two different onsets, no sharing) might influence the naming latencies continuously. Hence, the RT difference between the PO and the O conditions is not exactly the facilitation effect of preparing the single predictable onset, but instead a difference between this facilitation effect and the other facilitation effect of attending to two onsets. In this case, our results suggest that the PO condition generated a significantly stronger facilitation effect than the O condition did (still called “onset predictability effect” in the text below). Thus, the production behaviours of Chinese speakers were sensitive to our manipulation at single-segment level. In addition, the non-significant difference between the PR and the R conditions is consistent with the assumption of the attentional account that activation of a word-final component does not facilitate the encoding process. Otherwise we should have observed a similar difference between these two conditions as that between the PO and the O conditions.

### Theoretical implications

Previous studies in Chinese spoken word production have shown the behavioural effects of phonological overlap of more than one segment [12, 14–17], but evidence for the behavioural effect of single-segment overlap is lacking. The current study investigated the effects of segment predictability and segment repetition separately, which were mixed in previous form-preparation studies. An onset predictability effect was uncovered after the inhibition tendency of onset repetition was eliminated, filling the above research gap. This finding provides essential support to the claim that phonemic segments are functionally engaged in Chinese spoken word production. The functional role of segments in such a non-alphabetic language suggests that segments could be universal phonological units in spoken word production, as either proximate units or subordinate units.

Moreover, the current study showed a non-significant effect of rhyme predictability in contrast to the facilitation effect of onset predictability. This pattern, although not conclusive, adds to the little existing evidence [18] that is more compatible with serial encoding than parallel encoding of segments in Chinese spoken word production. The characteristics of Chinese language system may easily lead people to form an intuitive prediction that during spoken word production, segments within a Chinese syllable are encoded in parallel (e.g., [38]). Specifically, unlike in Germanic languages, phonemic segments are not orthographically represented in written Chinese. Each Chinese logogram corresponds to a syllable with clear boundaries, and the number of legitimate syllables is relatively limited. In addition, without the need of resyllabification (e.g., the English phrase *got it* becomes “go-tit” after resyllabification), it seems possible for Chinese speakers to store positional information intrinsically in segmental representations and to retrieve these position-specific segments within a syllable simultaneously. This possibility becomes even more plausible when considering the relatively uniform structure of Chinese syllables (e.g., lacking consonant clusters). That’s why serial encoding of segments in Chinese spoken word production, if proven to be true in future studies, would provide critical support to the universal aspect of spoken word production across different languages. At present, our finding is not conclusive, since the non-significance of the rhyme predictability effect might be real or indeed due to the limited sensitivity of the current design and measurement (just like the failure to find an onset effect in previous studies). More studies in Chinese as well as other languages are needed to find out whether serial encoding of segments in spoken word production could be universal across different languages.

Last but not least, the reinterpretation of the null onset effect in the Chinese form-preparation paradigm raises a question as to whether the cancellation account applies to the null tone effect in Chinese as well [36]. While Zhang found a null effect of tone sharing [36], Chen et al. obtained a 6-ms inhibition effect of tone sharing over three subexperiments [11]. Whether this suprasegmental inhibition effect is real or not remains unresolved. Hence, it might be helpful to examine the effects of tone repetition and tone predictability separately, like in the current study. Moreover, segmental predictability and suprasegmental predictability can also be factorially manipulated to investigate the potential interaction between segmental and suprasegmental processing in spoken word production (e.g., [14, 41]).

## Conclusions

In the form-preparation paradigm, Chinese speakers were able to make use of onset predictability and to prepare for naming responses. The onset facilitation effect was masked by the inhibition tendency of onset repetition, accounting for the overall null effect of onset overlap in previous Chinese form-preparation studies.

## Supporting information

S1 Data Raw data of naming latencies and accuracies. (XLSX)

S1 Text Picture sets in each type of context. (DOCX)

S2 Text Models in the LMEM analyses. (DOCX)
