# Supplementary material for: Behavioural evidence for segments as subordinate units in Chinese spoken word production: The form-preparation paradigm revisited
Source: PLoS One. 2019 Nov 27;14(11):e0225718. doi: 10.1371/journal.pone.0225718 (PMC6880989; doi:10.1371/journal.pone.0225718)
Supplement: S1 Text — (DOCX) [file pone.0225718.s002.docx]

Supporting Information for *PLoS One*

**S1 Text. Picture sets in each type of context.**

The sixteen pictures used in the current study are *糕* /gao1/ “*cake*”, *钩* /gou1/ “*hook*”, *柜* /gui4/ “*cabinet*”, *鸽* /ge1/ “*dove*”, *勺* /shao2/ “*spoon*”, *手* /shou3/ “*hand*”, *水* /shui3/ “*water*”, *蛇* /she2/ “*snake*”, *号* /hao4/ “*trumpet*”, *猴* /hou2/ “*monkey*”, *灰* /hui1/ “*gray*”, *盒* /he2/ “*box*”, *巢* /chao2/ “*nest*”, *愁* /chou2/ “*sorrow*”, *锤* /chui2/ “*hammer*”, *车* /che1/ “*car*”. Table A shows how they were grouped to form different contexts in the two experiments.

**Table A. Sets of picture names (in Pinyin) in each type of context.**

| Context | Set 1 | Set 2 | Set 3 | Set 4 |
| --- | --- | --- | --- | --- |
| Predictable onset repetition | /gao1/, /gou1/, /gui4/, /ge1/ | /shao2/, /shou3/, /shui3/, /she2/ | /hao4/, /hou2/, /hui1/, /he2/ | /chao2/, /chou2/, /chui2/, /che1/ |
| Predictable rhyme repetition | /gao1/, /shao2/, /hao4/, /chao2/ | /gou1/, /shou3/, /hou2/, /chou2/ | /gui4/, /shui3/, /hui1/, /chui2/ | /ge1/, /she2/, /he2/, /che1/ |
| Distributed segmental overlap | /gao1/, /chao2/, /gui4/, /chui2/ | /shao2/, /hao4/, /shui3/, /hui1/ | /gou1/, /chou2/, /ge1/, /che1/ | /shou3/, /hou2/, /she2/, /he2/ |
| Unrelated control | /she2/, /hui1/, /gao1/, /chou2/ | /shao2/, /hou2/, /gui4/, /che1/ | /gou1/, /chao2/, /shui3/, /he2/ | /ge1/, /chui2/, /shou3/, /hao4/ |
| Unpredictable onset repetition | /gao1/, /gui4/, /shou3/, /she2/ | /chao2/, /chui2/, /hou2/, /he2/ | /shui3/, /shao2/, /chou2/, /che1/ | /hui1/, /hao4/, /gou1/, /ge1/ |
| Unpredictable rhyme repetition | /gao1/, /chao2/, /shou3/, /hou2/ | /gui4/, /chui2/, /she2/, /he2/ | /shui3/, /hui1/, /gou1/, /chou2/ | /shao2/, /hao4/, /ge1/, /che1/ |
