# Supplementary material for: Behavioural evidence for segments as subordinate units in Chinese spoken word production: The form-preparation paradigm revisited
Source: PLoS One. 2019 Nov 27;14(11):e0225718. doi: 10.1371/journal.pone.0225718 (PMC6880989; doi:10.1371/journal.pone.0225718)
Supplement: S2 Text — (DOCX) [file pone.0225718.s003.docx]

Supporting Information for *PLoS One*

**S2 Text. Models in the LMEM analyses.**

Table B lists the formulas of different models in the LMEM analyses and the comparisons carried out among these models.

**Table B. Formulas and model comparisons in the LMEM analyses.**

|  | Model formulas and AICs | Model comparisons | | Effects being tested |
| --- | --- | --- | --- | --- |
| Expt 1 | m11: [invRT ~ cond*cyc + ord + (1 + cond \| participant) + (1 + cond \| picture)]  AIC = -344.71 | |  |  |
|  | m12: [invRT ~ cond + cyc + ord + (1 + cond \| participant) + (1 + cond \| picture)]  AIC = -348.35 | | anova(m11, m12) | The cond*cyc interaction |
|  | m13: [invRT ~ cyc + ord + (1 + cond \| participant) + (1 + cond \| picture)]  AIC = -345.08 | | anova(m12, m13) | The main effect of cond |
| Expt 2 | m21: [invRT ~ cond*cyc + ord + (1 + cond \| participant) + (1 + cond \| picture)]  AIC = -149.59 | |  |  |
|  | m22: [invRT ~ cond + cyc + ord + (1 + cond \| participant) + (1 + cond \| picture)]  AIC = -156.84 | | anova(m21, m22) | The cond*cyc interaction |
|  | m23: [invRT ~ cyc + ord + (1 + cond \| participant) + (1 + cond \| picture)]  AIC = -150.84 | | anova(m22, m23) | The main effect of cond |
| *Note*. cond: priming condition; cyc: cycle; ord: superblock order. | | | | |
